# Charting the Future of Stereoselective Carbohydrate Synthesis: The Essential Role of Cross‐Disciplinary Innovation

**DOI:** 10.1002/advs.76520

**Published:** 2026-07-13

**Authors:** Charles C. J. Loh

**Affiliations:** ^1^ UCD School of Chemistry University College Dublin, Belfield Dublin 4 Ireland

**Keywords:** carbohydrates, catalysis, glycosylation, noncovalent interactions, stereoselectivity

## Abstract

Cross‐disciplinary endeavours are well recognized to be incubators and propellors for frontier advancements. Established scientific fields often emerge out of commonality of research interests, and the need to consolidate knowledge through interactive exchanges within a commonly identified arena. Counter‐intuitively, there is a rapid paradigm shift for mining new knowledge through the willingness of crossing established fields. Paradoxically, when seemingly irrelevant chemistry fields are knitted together through their commonality in bond‐forming chemical phenomena, fresh new ideas to tackle long‐standing challenges emerge along the boundary lines. In this perspective dedicated to the Advanced Science 2025 Young Innovator Award, I will narrate my research group and my personal exciting journey in the discovery of knowledge at the intersection of numerous fields of catalysis, carbohydrate chemistry, and supramolecular chemistry/non‐covalent interactions. By sharing our laboratory's story in the discovery of the σ‐hole based catalytic glycosylation strategy, we hope this rewarding experience will provide the sparks for younger researchers to innovate beyond field‐specific paradigms. As researchers return to curiosity, inquisitiveness, and the pure enjoyment for chemistry, I believe that the assimilation of an interdisciplinary research paradigm into catalytic carbohydrate synthesis will serve as an impetus for the discovery of novel chemical phenomena throughout the chemical sciences.

## Graphical abstract

Cross‐disciplinary endeavors are blossoming across the broad chemical sciences. This perspective provides a glimpse into the rewarding interdisciplinary journey taken by the Charles Loh research group in pioneering the σ‐hole based catalytic glycosylation strategy. The author describes how the merger of catalysis, supramolecular, and carbohydrate chemistry, as well as the open‐mindedness in embracing diverse domains, is key in chemical discovery.

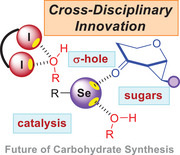

## 1 Introduction

**FIGURE 1:**
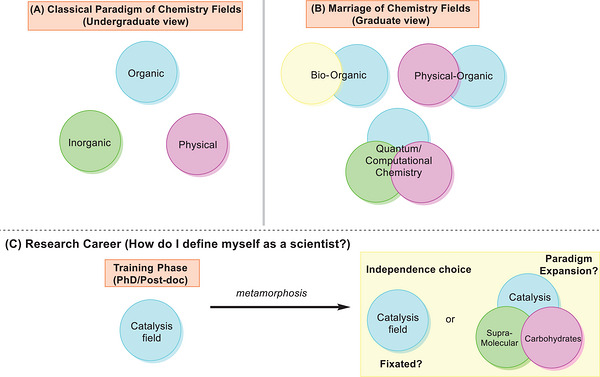
Scientific paradigms in chemistry and the choice of research paradigm upon independence.

A paradigm is a lens in which scientists can collectively appreciate, comprehend, and systemize knowledge. From the days of an undergraduate chemistry majors, we were educated into the classical paradigm that chemistry is firmly demarcated along the three pillars of physical chemistry, inorganic chemistry and organic chemistry (Figure 1A). This is definitely a useful paradigm that had shaped chemistry departments worldwide and determined research fields and trained countless chemists for both industry and academia. As one progresses toward graduate studies, the classical lines start to magically appear somewhat nuanced when graduate students start to encounter hybrid terminologies like “bioorganic chemistry,” “physical organic chemistry,” “medicinal chemistry” or “quantum/computational chemistry” (Figure 1B). The apparent blurring of boundaries at first glance is underpinned by the very fact that scientific knowledge is not static, and advancement in science is rooted upon a strong interdisciplinary essence. With ever new domains being coined by the marriage of conventional scientific arenas, it is fair to suggest that, unlike our traditional undergraduate paradigm, a dynamic intermingling of scientific knowledge is existential for scientific progress. This innovative power of cross‐domain conceptual pollination, known as the “*Medici effect*,” is well‐established in the entrepreneurship domains [1], but is substantially less appreciated in contemporary chemistry research.

I have always considered myself a classical organic chemist, loved organic chemistry lectures, and gained post‐graduate and post‐doctoral experiences in laboratories with a strong synthetic organic chemistry and catalysis focus. A significant shift occurred as I started my independent career in 2016 (Figure 1C), when I started to explore fields that were very foreign to me, in particular, in the fields of supramolecular and carbohydrate chemistry. Despite having no formal training in carbohydrate chemistry research, I am fascinated by the homochiral molecular structures of carbohydrates, which resonate deeply due to my expertise in asymmetric catalysis—primarily in the creation of chirality from prochiral substrates.

**FIGURE 2:**
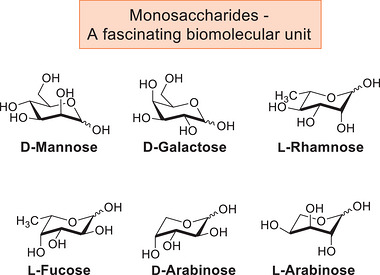
Molecular structure of common monosaccharides and their homochirality.

Carbohydrates are undoubtedly unique, both from the perspective of a specialist field [2, 3, 4] and as a molecular substrate class (Figure 2). Carbohydrates are homochiral [5], and found in nature predominantly in their d‐sugar forms, with some exceptions such as rhamnose and arabinose. For long, organic chemists with more classical catalysis training tend to shy away from carbohydrates due to their perceived structural sophistication and difficulties in handling. Hence, there is a common perception in the synthetic arena that the chemistry of carbohydrates should be better left to the specialists’ hands instead [3, 4, 6, 7].

After independence, I quickly realized that most researchers involved in carbohydrate chemistry had received carbohydrate chemistry training since their graduate research days. Crossing from the catalysis realm into glycochemistry is admittedly not a well‐trodden path, as sugars are generally viewed as overly sophisticated substrates that are better left to the carbohydrate specialists. Nevertheless, the fascinating molecular structure and the long‐standing inherent challenges in stereoselective carbohydrate chemistry nudged me to move beyond the conventional divides and to contribute broadly as an inquisitive chemist.

Most importantly, the belief that the commonality in improving the precision of bond‐forming pathways across the fields of catalysis, carbohydrate chemistry, and supramolecular chemistry could be a unique entry point for the discovery of unconventional carbohydrate synthesis protocols kept my enthusiasm ignited. This is the backdrop that led to our discovery of spectacular catalytic reaction modes when σ‐hole based catalysis merges with carbohydrate chemistry. The author hopes that our following narrative of the unexpected beginnings of the σ‐hole‐based catalytic glycosylation strategy could serve as an inspiration for carbohydrate and organic chemists to open new horizons in the current new era of catalytic carbohydrate synthesis.

## 2 The Early Roots on How the σ‐Hole Based Catalytic Glycosylation Strategy Budded

**FIGURE 3:**
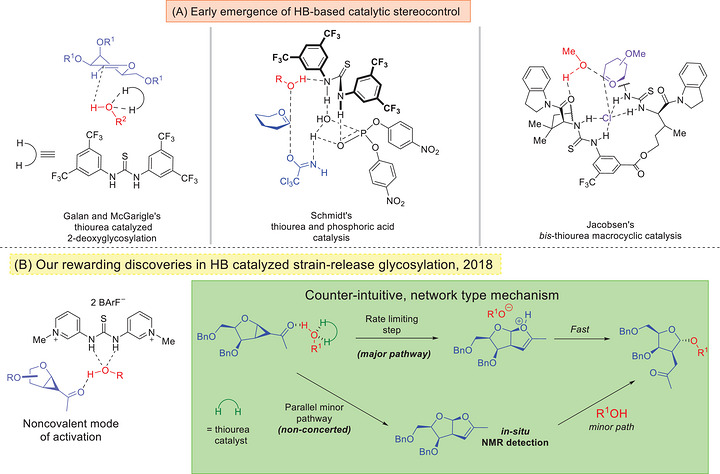
Precedents in HB‐catalyzed glycosylations, and the maiden discovery of a complex network‐like mechanism in strain‐release glycosylations during the author's early career phase.

My independent group initiated by embarking on thiourea‐catalyzed glycosylations in 2016 – an area inspired by early works from other prominent researchers such as Jacobsen [8], Galan [9], Ye [10] and Schmidt [11], who showed that harnessing hydrogen‐bonding interactions catalytically can be an efficient means of activating glycosyl substrates and rigidifying stereocontrolling transition states (Figure 3A). This led to our initial maiden publication in charged thiourea‐catalyzed strain‐release glycosylation reactions under extremely low catalyst loadings (Figure 3B) [12]. We also counter‐intuitively discovered an intriguing network‐type mechanism involving two parallel pathways, which further piqued our interest into dissecting hitherto unknown pathways embedded in carbohydrate chemistry. These initial reports in the field serve as a prelude that noncovalent interactions (NCIs) could play a central role in dictating stereoselectivity in glycosidic bond‐forming events.

**FIGURE 4:**
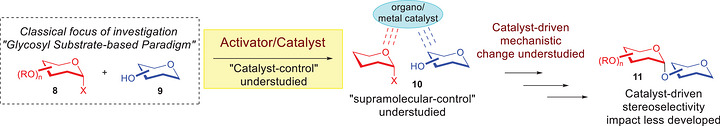
Mainstream focus on investigating glycosyl substrates versus less explored domains in catalyst‐control, supramolecular‐control, and catalyst‐driven mechanistic changes.

However, it is important to mention that the recognition of NCIs as a conceptual pillar in biasing glycosylation selectivity [13, 14] and the general realization of catalytic supramolecular events that led to beneficial stereochemical outcomes in carbohydrate chemistry [15] was virtually non‐existent at that juncture. The predominant mainstream view was still very much focused on casting the emphasis upon protecting group, conformational and the reactivity of the glycosyl substrates **8** and **9** – a classical perspective we termed as the *“glycosyl substrate‐based paradigm”* (Figure 4) [13, 16].

Notwithstanding, we were driven to question if non‐classical interactions could serve as alternative drivers beyond the substrate‐focused perspective, such as catalyst and supramolecular control that could steer and influence outcomes in the glycosylation mechanism to precisely construct glycosidic linkages to form **11** [17]. It was in 2017 that our group was made aware of nascent advances in halogen bonding (XB) catalysis [18, 19, 20], in particular those *bis*‐benzoimidazolium XB donors newly developed by the group of Stefan Huber [21]. Halogen bonding belongs to a larger family of unconventional interactions, known as σ‐hole based interactions that originated from the electronic anisotropic of group IV‐VII elements [22, 23, 24].

σ‐hole based interactions are fascinating, primarily because they involve the exploitation of concentrated electropositive regions on the electrostatic potential surface of elements such as halogens or chalcogens that are intuitively presumed to be electron‐rich. The “σ‐hole” terminology can be attributed to the localized electropositive region located on the antipode of the C‐X or C‐Ch covalent bond axis, or along the σ^*^‐orbital axis [17]. This geometrically defined and concentrated positive region forges highly directional interactions with electron donors, for instance, lone pairs on Lewis bases or π‐electrons. Besides the electrostatic component that is operative, these interactions also comprise of other components such as orbital delocalization, polarizability, dispersion alongside charge transfer [19, 22]. Furthermore, the identity of the σ‐hole donor element modulates the σ‐hole properties. The progression from the top to the bottom of a group in the periodic table (e.g. in group VII from bromine to iodine) results in the expansion of the σ‐hole size. Furthermore, switching from neutral halogen bonding (XB) to ChB (Ch(II) valency) donors results in a numerical increase in the number of electrophilic axes. Valency expansion further deepens the σ‐holes by lowering the energy of the σ^*^ orbital, together with the increase in the numerical quantity of the presented σ‐holes [17]. These unique σ‐hole properties offer unprecedented tunability to σ‐hole based catalysis that cannot be easily replicated via the classical hydrogen bonding (HB) catalytic congeners.

Intriguingly, the exploitation of such non‐classical interactions in catalysis was rare despite their rather unusual physical properties. Despite our general understanding as chemists that hydrogen bonding is a highly directional interaction, we were surprised to learn that the lesser mentioned σ‐hole based interactions possess even higher directionality than their HB congeners [19, 22, 25, 26, 27]. Moreover, there was virtually no knowledge of how these non‐protic‐based weak interactions could impact carbohydrate synthesis and lead to desirable outcomes in glycosylations [15], apart from an early proof‐of‐principle unselective Königs‐Knorr glycosylation promoted by stoichiometric quantities of an XB promoter by Huber and Codée [28]. These knowledge gaps further piqued our interest to move beyond the boundaries of these segregated fields and explore new knowledge that could simultaneously benefit these fields concurrently.

**FIGURE 5:**
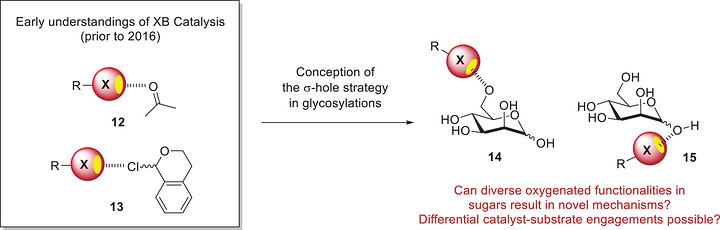
Early conception of the potential of σ‐hole activation on glycosyl substrates.

Learning from the stellar development of thiourea catalysis [29, 30, 31, 32, 33], where molecules known in supramolecular host–guest studies can be transposed into the catalysis realm, we were excited with the prospect of catalytically leveraging the non‐protic, “soft,” higher directionality and electrophilic axis tunability in carbohydrate activation. Benefiting from early proof‐of‐concept cases that were generally restricted to more conventional carbonyl (see **12,** σ‐hole denoted in yellow oval) and alkyl halide activation (see **13**), we envisioned that the multiple spatially distinctive oxygen moieties embedded within saccharides may uniquely engage with σ‐hole donor catalysts in highly directional modes such as **14** or **15** unachievable using conventional hydrogen‐bonding, Lewis acid or Brønsted acid based catalytic modes (Figure 5). We were also enthusiastic in pushing the limits of σ‐hole interactions and glycochemistry, and to explore if new synergies could arise from interfacing seemingly unrelated concepts segregated in different scientific communities.

**FIGURE 6:**
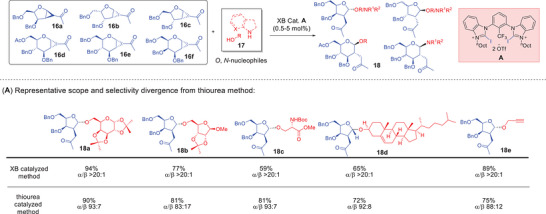
Our seminal XB‐catalyzed strain‐release glycosylation [34].

Our approach was rewarded in 2018, when we discovered that XB‐catalyzed strain‐release glycosylation was consistently yielding excellent α:β anomeric selectivity over the entire substrate scope [34]—an observation that was certainly not replicable using our prior protocol that taps upon thiourea catalysis [12], which yielded inconsistent stereoselectivity (Figure 6). We learned from this experience that in the scientific discovery process, a healthy balance has to be struck between rationality and the tolerance of uncertainty. While science is built upon rational principles, the events that lead up to major discoveries often possess a serendipitous element. We were hence driven by the possibility of an unexpected XB‐catalyzed mechanism, and to study the molecular basis of this surprising stereoselectivity.

**FIGURE 7:**
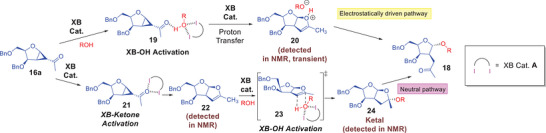
The unexpected unraveling of reversible iterative σ‐hole ···O catalytic interactions in XB‐catalyzed strain‐release glycosylation [34].

By using physical organic chemistry approaches, such as kinetics and in situ NMR detection of intermediates, together with meticulous control experiments, we were able to propose a working mechanistic hypothesis where XB catalytic activation can be proven for the first time to be operative in multiple elementary steps on the reaction coordinate (Figure 7). This is a fascinating reaction network that comprises of two parallel pathways; one that involved an electrostatically driven pathway through ionic intermediate **20**, and another simultaneous route whereby intermediates **22** and **24** are involved. This mechanistic postulate was also supported by in situ ^1^H NMR monitoring of the reaction. This discovery was significant in 2019 as it led to a central mechanistic understanding that reversible σ‐hole interactions can be harnessed in complex mechanistic settings to yield favorable reactivity and stereoselectivity outcomes.

## 3 From XB‐Catalyzed Glycosylation Discovery to Conceptual Expansion Towards Other Glycosidic Platforms

The momentum grew as we realized that the reversible XB catalytic phenomena was not simply restricted to the strain‐release glycosylation platform. We were well aware in 2018 that there was a substantial interest in the elegant 2‐deoxyglycosylation [35] from glycals by various colleagues worldwide [9, 36, 37, 38]. Furthermore, there was a prevailing view that general Lewis/Bronsted acid activation by promoters or catalysts were functionally equivalent to newer catalytic modes in activating glycals, and the mechanistic understanding in this 2‐deoxyglycosylation domain was presumably established by analogy. Nevertheless, upon careful scrutiny of examples that were present in the literature, we discovered that subtle substrate‐based caveats exist. For instance, the seminal Schreiner thiourea‐catalyzed 2‐deoxyglycosylation was only reported for the galactal substrate [9]. Handling‐wise, protocols using thiourea‐type catalysis were cumbersome, as these glycosylations require stringent moisture removal [9, 39, 40]. These were tangible but less discussed aspects to improving the tractability of such reactions for non‐carbohydrate chemists.

**FIGURE 8:**
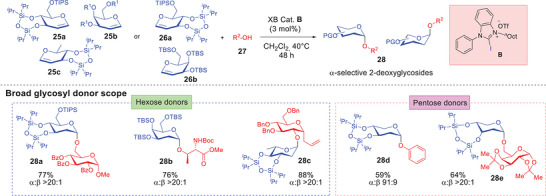
Expansion of the XB‐catalyzed glycosylation strategy to 2‐deoxyglycosylations.

Spurred by the hypothesis that σ‐hole‐based activation could be mechanistically distinctive compared to HB activation, we counter‐intuitively discovered that an XB‐catalyzed 2‐deoxyglycosylation of natural and non‐natural glycals **25**, **26** performed more robustly than previously reported thiourea and thiouracil congeners (Figure 8) [41]. The water tolerance aspect was significantly more robust, as reported prolonged high vacuum treatment of substrates prior to 2‐deoxyglycosylations was not required [9, 39, 40].

Meticulously performed bench‐marking experiments that reproduced prior thiourea/thiouracil catalyzed cases gave us valuable insights into the broader substrate tolerance of our XB‐catalyzed 2‐deoxyglycosylation. For instance, other natural glycosidic substrates such as glucal, rhamnal, and arabinals could be better tolerated in our XB‐catalyzed strategy.

**FIGURE 9:**
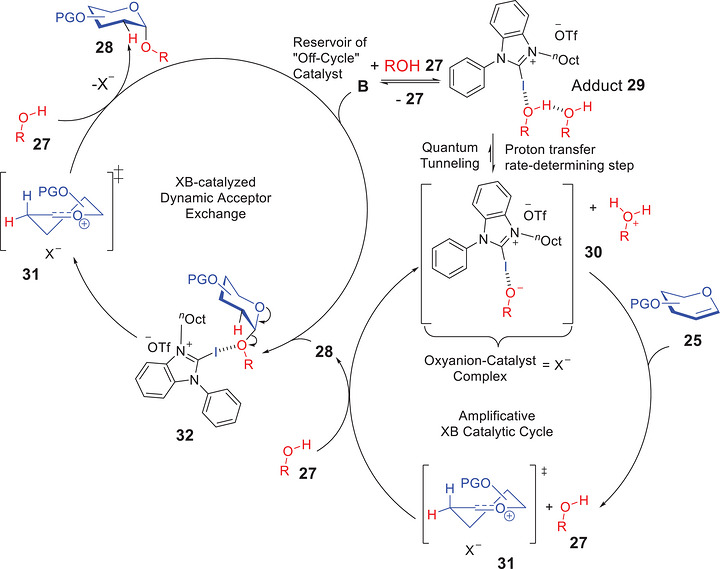
Mechanistic proposal of the XB‐catalyzed 2‐deoxyglycosylation that once again revealed reversible XB···O interactions in a complex mechanistic network [41].

Importantly, our mechanistic studies revealed that the iterative XB catalytic activation mechanism discovered in our previous study was also in operation (Figure 9). Through a judiciously selected suite of mechanistic experiments that involved NMR monitoring, kinetics and NMR titrations, we brought about evidence that a plethora of halogen‐oxygen interactions were operative between the XB catalyst and the glycosyl acceptor's hydroxyl group in **29** as well as the oxyanion in **30**. Counterintuitively, there was an XB interaction between the catalyst and the newly forged glycosidic linkage in **32**, which resulted in a dynamic glycosidic linkage cleavage and reforming of that same linkage by a new molecule of nucleophile. These intricacies were observed in ^1^H NMR monitoring of the 2‐deoxyglycoside product with a different nucleophile (propargyl alcohol) in the presence of catalyst **B**. This led to the proposal of a mechanism that involved multi‐step XB activation as well as the involvement of quantum tunneling [42, 43] in the rate‐limiting step.

## 4 From Multi‐Step XB‐Catalyzed Glycosylations to Mechanistically Novel ChB‐Catalyzed Glycosylations

**FIGURE 10:**
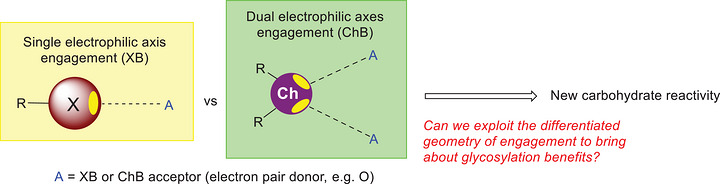
The numeric differences in σ‐holes between neutral XB and ChB donors as an exploitable phenomenon to access novel σ‐hole based glycosylation mechanisms.

Encouraged by our unusual observations in XB‐catalyzed glycosylations, we turned to question if “*sister interactions*” of the σ‐hole family, such as chalcogen bonding (ChB) interactions, could operate in mechanistically distinctive ways [17, 24, 44, 45, 46]. Besides the literature knowledge that neutral chalcogen (II) donors are endowed with two σ‐holes along the antipode of the C─Ch bond axis—a geometric difference compared to the one σ‐hole available on neutral XB donors, almost nothing was known on how these differences in the quantity of electrophilic axes could be translated into reactivity or stereoselectivity benefits in carbohydrate synthesis. We hence wondered if the dual electrophilic axes of engagement imparted by ChB donors (Figure 10) could be exploited for the discovery of novel carbohydrate reactivity, since this differs fundamentally from the single electrophilic engagement mode we previously pursued, which was possible with XB donors.

We were also intrigued that when we began to explore this domain in the early 2020s, there was virtually no literature precedent in the use of ChB catalysis [19, 26] in carbohydrate chemistry [15, 17, 44, 45, 46, 47]. Inspired by modularly designed phosphonochalcogenide (PCH) catalysts developed by the Wang group in 2019 that were easy to synthesize [48], we explored the possibility of harnessing ChB catalysis to access novel glycosidic pathways. We posit that new mechanistic pathways have the potential to lead to unique stereoselectivity and reactivity outcomes. Our breakthrough occurred when we were investigating a largely underexplored class of glycomimetics known as septanosides. These are 7‐ring sugar scaffolds that contain a privileged oxepane core. Oxepane motifs are often found in marine natural products [49], as well as in other bioactive molecules [50, 51, 52, 53, 54]. Despite these interesting traits, prevailing methods in 2020 to access septanosides were largely restricted to entropically unfavorable macrocyclizations [55, 56, 57, 58]. We were thus very interested to explore an alternative strain‐release ring expansion approach which offers a thermodynamically favorable route into such highly prized scaffolds. However, previous attempts by Hoberg revealed immense stereoselectivity and substrate‐dependency challenges in strain‐release septanosylations [59, 60].

**FIGURE 11:**
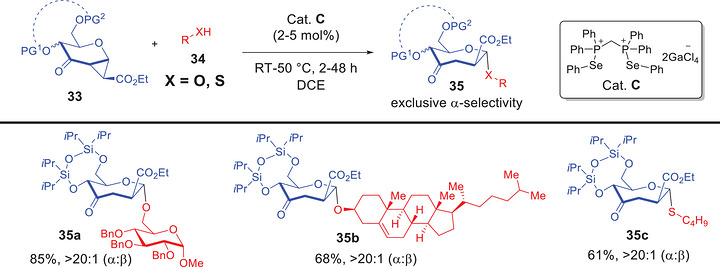
Seminal report of a ChB‐catalyzed glycosylation to access *O*,*S*‐septanosides [61].

**FIGURE 12:**
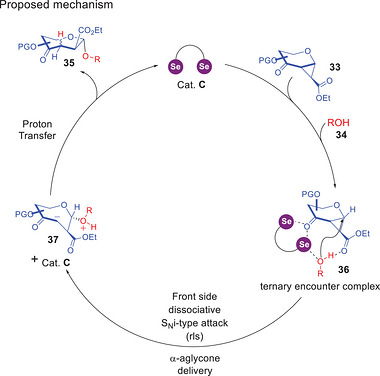
Proposed interplay of bifurcated ChB and HB in the septanosylation stereocontrol.

Unexpectedly, when we employed PCH catalyst **C** on a cyclopropanated substrate **33** in the presence of a model *O*‐acceptor, we noted an outstanding catalytic performance at 2 mol% catalyst loading, generating the target septanoside **35** with exclusive α‐selectivity (Figure 11) [61]. Furthermore, the scope was vast and assimilates *O*‐ and *S*‐nucleophiles in varying steric environments. As we delved into the mechanistic intricacies of this reaction, we were pleasantly surprised that an unexpected mode of bifurcated ChB activation that involved the simultaneous engagement of both σ‐holes of the selenium atom was operative in **36**. Furthermore, the synergistic interplay of ChB and HB in the S_N_i‐type nucleophilic attack in the rate‐limiting step to form **37** was found to be instrumental in dictating the excellent α‐selectivity outcome (Figure 12).

**FIGURE 13:**
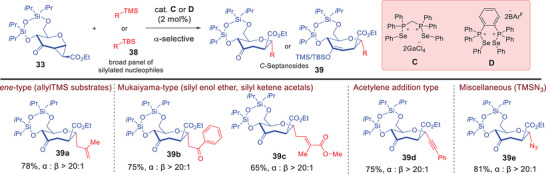
ChB‐catalyzed *C*‐septanosylation [62].

**FIGURE 14:**
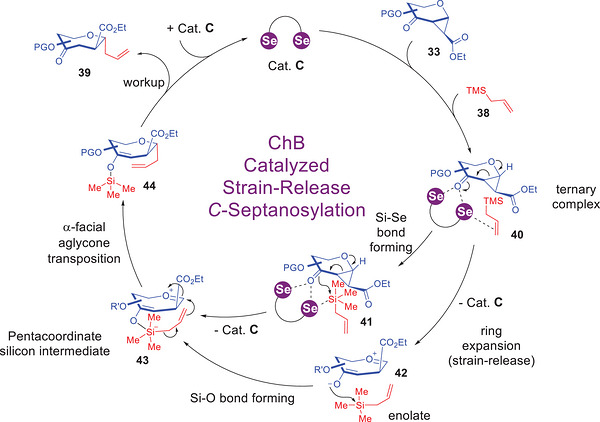
Mechanistic switch in *C*‐septanosylations [62].

The mechanistic surprises associated with σ‐hole based activation on glycosyl substrates continued. By simply switching the nucleophile from protic hydroxyl/thiol based acceptors to silylated non‐protic *C*‐acceptors **38**, we observed that an array of carbon‐based synthons can be easily installed onto septanosides to yield the highly demanded *C*‐septanoside (α,α’‐*C*‐disubstituted oxepanes) scaffold **39** (Figure 13) [62]. The more intriguing aspect is that the nucleophile switch resulted in a mechanistic switch towards an intramolecular aglycone transposition from a pentacoordinate silicon intermediate (Figure 14) [63, 64]. Hence, the mechanistic outcome of chalcogen bonding‐based glycosylation was found to be sensitive to the nucleophile choice.

**FIGURE 15:**
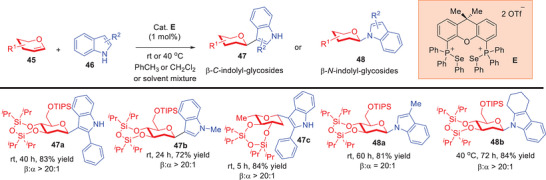
ChB‐catalyzed β‐*C*,*N*‐indolyl glycosylation [70].

In our investigation to seek new synthetic routes into the prevalent *C*‐, *N*‐β‐indolyl glycoside scaffold that possess anti‐diabetic [65, 66] and anti‐inflammatory activities [67], we were exploring new catalytic methods that could facilitate the direct construction of indolyl‐glycosides **47, 48** from easily available glycals **45** and indoles **46**. Appreciating the divergent reactivity enforced by the multi‐nucleophilic sites on the C3 and N1 of indoles [68, 69], we surmise that a robust strategy that concomitantly enables versatile access into both *C*‐ and *N*‐indolyl glycosides would be highly valuable. We were pleasantly surprised that when PCH catalyst **E** was employed in this endeavor, it enabled β‐selective access into *C*‐indolyl and *N*‐indolylglycosides **47** and **48** (Figure 15) [70].

**FIGURE 16:**
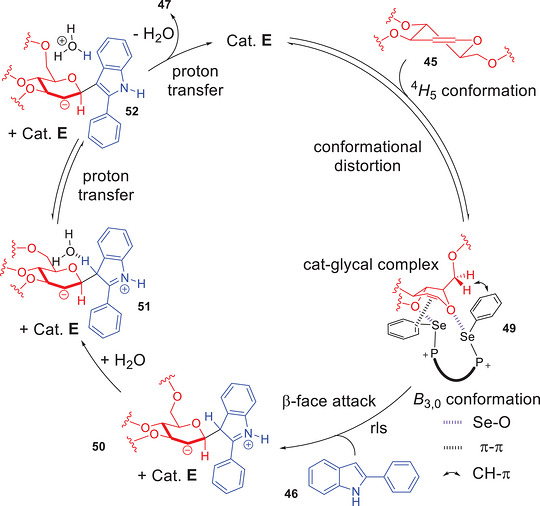
Mechanism of the ChB‐catalyzed β‐*C*‐indolyl glycosylation that involves conformational distortion [70].

Mechanistic studies yielded exciting noncovalent insights that support the uniqueness of PCH catalyst engagement on glycosyl substrates. We noted for the first time that the aromatic flanks of PCH catalysts can also be tapped upon catalytically apart from the ChB contributions from the seleniums. Furthermore, through NMR titrations and DFT calculations, we brought forth evidence that both π‐interactions and Se···O ChB interactions can collectively distort the half‐chair to a boat conformation in **49** to facilitate a highly β‐selective nucleophilic attack from the convex β‐face of the glycal (Figure 16).

**FIGURE 17:**
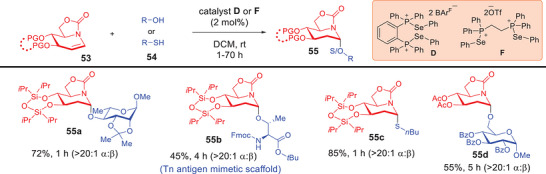
ChB‐catalyzed α‐iminoglycosylation [75].

Riding on the momentum of this series of ChB catalytic discoveries, we expanded our investigation to an underexplored class of biologically relevant glycomimetics known as iminosugars [71, 72, 73, 74]. These aza‐mimics of pyranosides are generally less explored in carbohydrate synthesis, but of immense biological and synthetic interest. By employing PCH catalysts **D** and **F**, we discovered a robust *O*‐iminoglycosylation of sp^2^‐iminoglycals **53** [73] that exclusively yielded α‐iminoglycosides **55** (Figure 17) [75].

**FIGURE 18:**
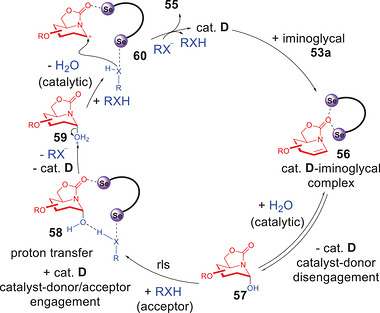
Multi‐elementary step ChB activation manifold demonstrated in iminoglycosylations [75].

It is pertinent to also mention that this robust strategy is highly moisture tolerant and proceeded smoothly even under ambient conditions without rigorous water exclusion seen in alternative catalytic strategies [76]. The reaction manifold surprisingly harnesses trace water catalytically in its elementary steps to yield the water addition intermediate **57**, which was isolated and characterized. Control experiments also ascertained that **57** can only react with the glycosyl acceptor in the presence of catalyst **D** to yield **55a**. Remarkably, we confirmed in this protocol that the iterative reversible σ‐hole activation strategy across multiple elementary steps previously observed in our XB‐catalyzed glycosylations [34, 41] was also amenable in ChB catalyzed iminoglycosylations (Figure 18). This realization offers a further validation that our discovered reversible iterative σ‐hole activation mechanism is a general phenomena across different types of σ‐hole based catalysis.

Having established in the fore‐mentioned methods that ChB‐based PCH catalysis can bring about unique mechanistic manifolds that were irreplicable using XB or thiourea catalysis, our team was confronted with a fundamental question of why there are differentiated catalytic outcomes across XB and ChB catalysis despite their commonality in the σ‐hole activation phenomena. Recently, this question was put to the test in a puzzling observation in our seminal XB‐catalyzed strain‐release glycosylation [34]. Back in 2019, we unexpectedly noted that XB catalysis only enabled stereoselective glycosylations in *O*‐ and *N*‐glycosylations but not in aryl‐*C*‐glycosylations. Appreciating that aryl‐*C*‐glycosides are important structural scaffolds in anti‐diabetic agents, we wondered whether fascinating advances in ChB catalysis could open up favorable new mechanistic pathways to access aryl‐*C*‐glycosides stereoselectively.

**FIGURE 19:**
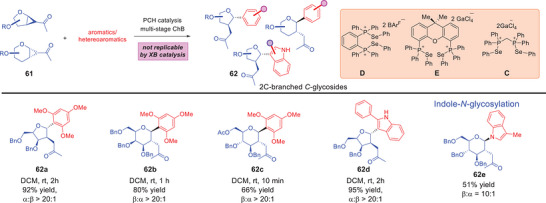
An exclusively ChB‐catalyzed *C*‐strain‐release glycosylation that is irreplicable by the conceptually similar XB catalysis [77].

**FIGURE 20:**
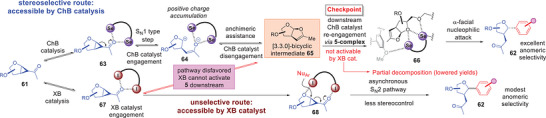
Mechanistic rationale of stereoselectivity divergences between ChB and XB‐catalyzed *C*‐strain‐release glycosylations [77].

Gratifyingly, we discovered a highly stereospecific ChB‐catalyzed aryl‐*C*‐strain‐release glycosylation (Figure 19) that assimilates electron‐rich aromatics and heteroaromatics [77]. In direct contrast, the XB‐catalyzed version of this reaction yielded substantially diminished stereoselectivity and yields. Furthermore, mechanistic studies that involves NMR titration, poisoning experiments, Hammett analysis and DFT modeling of the catalyst‐intermediate‐nucleophile ternary complex provided evidence that the differentiated σ‐hole activation of a key bicyclic intermediate **65** through double bifurcated ChB interactions in ternary complex **66** underpins the ChB catalytic exclusivity in this strategy (Figure 20). Moreover, the Hammett analysis suggests that ChB catalysis shifted the reaction mechanism to the S_N_1 domain, while XB catalysis proceeded with an asynchronous S_N_2 type manifold through **68** with mild dissociative character. This work hence offered for the first time a mechanistic rationale to account for the divergent reactivity and stereoselectivity outcomes when different σ‐hole donors are employed as catalysts in glycosylations (Figure 20) [77].

## 5 Cross‐Disciplinary Innovations in Carbohydrate Synthesis Also Evident in the Nascent Field of Photoredox‐Catalyzed Glycosylations

Besides our enriching journey in merging multiple disciplines to accelerate discovery in the chemical and glycosciences, we realized that such field‐intersecting experiences are not exclusively unique to our research team. Similar developments are also occurring globally amongst the younger generation of chemists [16]. This is particularly evident in recent booming advances in the use of photoredox catalysis in glycosylations [78, 79, 80, 81, 82]. This trend can be attributed to the rise in the curiosity‐driven approach [83], where mainstream catalysis chemists are now actively engaging carbohydrate‐based substrates as an excellent driver to advance the discovery of new mechanistic phenomena [16].

**FIGURE 21:**
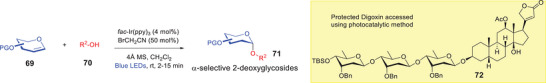
Cross‐disciplinary innovation merging photoredox catalysis and carbohydrate chemistry by Ye and co‐workers in iterative 2‐deoxyglycoside synthesis [86].

It is also essential in the discussion to mention elegant foundational photoinduced *O*‐glycosylation strategies reported by the group Ye using stoichiometric Cu(OTf)_2_ oxidant under UV irradiation [84, 85]. These pioneering work also led to their subsequent significant development in the facile photocatalyzed iterative synthesis of 2‐deoxyoligosaccharides using the *fac*‐Ir(ppy)_3_ photocatalyst under blue light irradiation (Figure 21) [86]. The reaction is highly robust and a plethora of 2‐deoxyglycosidic linkages can be easily accessed within minutes. Furthermore, this new strategy was further extended to the concise synthesis of protected digoxin **72** with good yields and selectivity.

**FIGURE 22:**
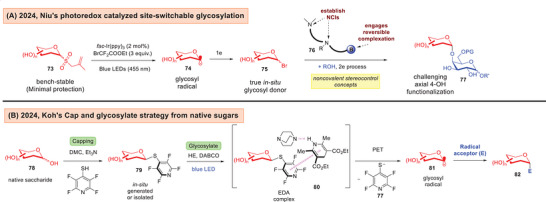
Cross‐disciplinary innovations in the interface between photoredox catalysis and carbohydrate chemistry by Niu and Koh [87, 88].

Other noteworthy recent advancements include Niu's use of bench‐stable, minimally protected allyl glycosyl sulfone donors **73** and acceptors in site‐switchable photoredox‐catalyzed glycosylations through a noncovalent stereocontrolling strategy (Figure 22A) [87]. The key of this protocol hinges in the design of a bifunctional organoboron catalyst of type **76** that can engage in reversible complexation with a diol in conjunction with a proximal amine that can establish noncovalent interactions with the sugar substrates. Another elegant work in combining the diverse fields of photoredox catalysis, noncovalent interactions and carbohydrate chemistry was reported in 2024 by Koh and Davis, where unprotected native saccharides **78** can be easily converted into a range of α‐*C*,*Se*,*S*‐glycosides through a “*cap and glycosylate”* strategy (Figure 22B) [88]. This method uses 2,3,5,6‐tetrafluoropyridine‐4‐thioglycoside for an in situ “traceless activation” to form **79**, which is followed up by the formation of a glycosyl radical **81**. **81** is finally be trapped by a radical acceptor **E** to yield α‐configured *C*‐glycosides **82**.

## 6 Conclusion and Outlook Regarding the Future of Stereoselective Carbohydrate Synthesis

Innovations in science often occur by traversing the classical confines of traditional fields, and embracing new (and arguably unfamiliar) ideas that arise from valuable insights buried in diverse scientific communities. Arriving at the nexus of scientific domains would therefore require intrinsic openness in appreciating the value of multi‐disciplinary collaborations, and moving beyond the standard line of questioning established in the individual fields.

Through our unique journey in the exploration of the σ‐hole based catalytic glycosylation strategy, as well as the concurrent emergence of novel synthetic domains in photoredox‐catalyzed carbohydrate synthesis, it is the author's wish that younger chemists could derive inspiration on how new research directions are forged by embracing intersectionality. Our “*Medici*” type paradigm is certainly not an isolated development, as upcoming intersectional directions involving the merger of supramolecular capsule catalysis with carbohydrate synthesis [89, 90, 91, 92], as well as recent interesting cross‐pollination between electrochemistry and carbohydrate chemistry [93, 94] promise cross‐disciplinary opportunities for cutting‐edge innovation.

It is my sincere belief that with tenacity and the willingness to embrace the uncertainty of interacting with multi‐fields, young chemists will be equipped with the readiness to consistently “learn and re‐learn.” This calls for a passionate academic spirit to innovate with joy in the ever‐changing scientific landscape. Moving beyond classical boundaries is immensely rewarding. It offers a freedom to explore solutions beyond the mainstream paradigms of a field, while opening expanded dimensions of solutions not classically available within the confines of a particular field.

## Funding

Research Ireland (Frontiers for the Future Awards), Boehringer Ingelheim Foundation (Plus 3 Perspectives Programme), Fonds der Chemischen Industrie (Liebig Fellowship, Li 198/04).

## Conflicts of Interest

The authors declare no conflicts of interest.

## Data Availability

Data sharing not applicable to this article as no datasets were generated or analysed during the current study.

## References

[1] Y. Lee , A. F. Cortes , A. Di Benedetto , et al., “The Medici Effect: Multidisciplinary Insights for Entrepreneurship Research,” New England Journal of Entrepreneurship 27 (2024): 2–8, 10.1108/NEJE-07-2024-104.

[2] T. J. Boltje , T. Buskas , and G. J. Boons , “Opportunities and Challenges in Synthetic Oligosaccharide and Glycoconjugate Research,” Nature Chemistry 1 (2009): 611–622, 10.1038/nchem.399. PMC2794050 20161474

[3] X. Zhu and R. R. Schmidt , “New Principles for Glycoside‐Bond Formation,” Angewandte Chemie International Edition 48 (2009): 1900–1934, 10.1002/anie.200802036. 19173361

[4] C. J. Crawford and P. H. Seeberger , “Advances in Glycoside and Oligosaccharide Synthesis,” Chemical Society Reviews 52 (2023): 7773–7801, 10.1039/D3CS00321C. 37830906

[5] S. Toxvaerd , “The Role of Carbohydrates at the Origin of Homochirality in Biosystems,” Origins of Life and Evolution of Biospheres 43 (2013): 391–409, 10.1007/s11084-013-9342-5. 23996458

[6] G. Xiao , “Glycosyl ortho‐(1‐Phenylvinyl)benzoates as Donors for Streamlined One‐Pot Assembly of Carbohydrates From Oligosaccharides to Polysaccharides,” Accounts of Chemical Research 58 (2025): 2350–2363, 10.1021/acs.accounts.5c00387. 40600889

[7] W. Yao and X. S. Ye , “Donor Preactivation‐Based Glycan Assembly: From Manual to Automated Synthesis,” Accounts of Chemical Research 57 (2024): 1577–1594, 10.1021/acs.accounts.4c00072. 38623919

[8] Y. Park , K. C. Harper , N. Kuhl , E. E. Kwan , R. Y. Liu , and E. N. Jacobsen , “Macrocyclic Bis‐Thioureas Catalyze Stereospecific Glycosylation Reactions,” Science 355 (2017): 162–166, 10.1126/science.aal1875. 28082586 PMC5671764

[9] E. I. Balmond , D. M. Coe , M. C. Galan , and E. M. McGarrigle , “α‐Selective Organocatalytic Synthesis of 2‐Deoxygalactosides,” Angewandte Chemie International Edition 51 (2012): 9152–9155, 10.1002/anie.201204505. 22887611

[10] L. Sun , X. Wu , D. C. Xiong , and X. S. Ye , “Stereoselective Koenigs–Knorr Glycosylation Catalyzed by Urea,” Angewandte Chemie International Edition 55 (2016): 8041–8044, 10.1002/anie.201600142. 27244701

[11] Y. Geng , A. Kumar , H. M. Faidallah , H. A. Albar , I. A. Mhkalid , and R. R. Schmidt , “Cooperative Catalysis in Glycosidation Reactions With O‐Glycosyl Trichloroacetimidates as Glycosyl Donors,” Angewandte Chemie International Edition 52 (2013): 10089–10092, 10.1002/anie.201302158. 23893796

[12] C. Xu and C. C. J. Loh , “An Ultra‐Low Thiourea Catalyzed Strain‐Release Glycosylation and a Multicatalytic Diversification Strategy,” Nature Communications 9, no. 1 (2018): 4057, 10.1038/s41467-018-06329-4. PMC6170412 30282986

[13] H. Guo and C. C. J. Loh , “Noncovalent Interactions: An Emerging Focal Point in Stereoselective Catalytic Carbohydrate Synthesis,” Carbohydrate Research 552 (2025): 109458, 10.1016/j.carres.2025.109458. 40132292

[14] C. C. J. Loh , “Exploiting non‐covalent interactions in selective carbohydrate synthesis,” Nature Reviews Chemistry 5 (2021): 792–815. 37117666 10.1038/s41570-021-00324-y

[15] A. T. Sebastian and C. C. J. Loh , “Emerging Capabilities of Nonclassical Noncovalent Interactions and Asymmetric Catalysis in Stereoselective Glycosylations and Carbohydrate Functionalizations,” Accounts of Chemical Research 58 (2025): 2124–2144, 10.1021/acs.accounts.5c00289. 40503847 PMC12224338

[16] C. C. J. Loh , “Catalytic Strategies for Stereoselective Carbohydrate Synthesis: Emerging Concepts for Accessing Challenging Glycosides,” Angewandte Chemie International Edition 64 (2025): 202514167. 10.1002/anie.202514167 PMC12518707 40944370

[17] H. Guo and C. C. J. Loh , “Nascent Stereocontrolling Approaches in σ‐Hole Based Catalysis,” Coordination Chemistry Reviews 551 (2026): 217447, 10.1016/j.ccr.2025.217447.

[18] D. Bulfield and S. M. Huber , “Halogen Bonding in Organic Synthesis and Organocatalysis,” Chemistry—A European Journal 22 (2016): 14434–14450, 10.1002/chem.201601844. 27465662

[19] D. Jovanovic , M. Poliyodath Mohanan , and S. M. Huber , “Halogen, Chalcogen, Pnictogen, and tetrel Bonding in Non‐Covalent Organocatalysis: An Update,” Angewandte Chemie International Edition 63 (2024): 202404823. 10.1002/anie.202404823 38728623

[20] R. L. Sutar and S. M. Huber , “Catalysis of Organic Reactions Through Halogen Bonding,” ACS Catalysis 9 (2019): 9622–9639, 10.1021/acscatal.9b02894.

[21] S. H. Jungbauer and S. M. Huber , “Cationic Multidentate Halogen‐Bond Donors in Halide Abstraction Organocatalysis: Catalyst Optimization by Preorganization,” Journal of the American Chemical Society 137 (2015): 12110. 26329271 10.1021/jacs.5b07863

[22] G. Cavallo , P. Metrangolo , R. Milani , et al., “The Halogen Bond,” Chemical Reviews 116 (2016): 2478–2601, 10.1021/acs.chemrev.5b00484. 26812185 PMC4768247

[23] A. Pizzi , G. Terraneo , C. L. Iacono , R. Beccaria , A. Dhaka , and G. Resnati , “Benefits of Categorizing Noncovalent Bonds Based on Hydrogen, Halogen, Chalcogen, and Pnictogen Bonds,” Angewandte Chemie International Edition 65 (2026): 6901593, 10.1002/anie.6901593. PMC13007575 41703764

[24] A. Pizzi , G. Terraneo , C. Lo Iacono , R. Beccaria , A. Dhaka , and G. Resnati , “Taxonomy of chemical bondings: Opportunities and challenges,” Angewandte Chemie International Edition 64 (2025): 202506525. 10.1002/anie.202506525 PMC12207382 40401347

[25] M. Breugst and J. J. Koenig , “σ‐Hole Interactions in Catalysis,” European Journal of Organic Chemistry 2020 (2020): 5473–5487, 10.1002/ejoc.202000660.

[26] G. Sekar , V. V. Nair , and J. Zhu , “Chalcogen Bonding Catalysis,” Chemical Society Reviews 53 (2024): 586–605, 10.1039/D3CS00503H. 38059482

[27] L. Vogel , P. Wonner , and S. M. Huber , “Chalcogen Bonding: An Overview,” Angewandte Chemie International Edition 58 (2019): 1880–1891, 10.1002/anie.201809432. 30225899

[28] R. Castelli , S. Schindler , S. M. Walter , et al., “Activation of Glycosyl Halides by Halogen Bonding,” Chemistry—An Asian Journal 9 (2014): 2095–2098, 10.1002/asia.201402259. 24962953

[29] S. J. Connon , “Organocatalysis Mediated by (Thio)urea Derivatives,” Chemistry—A European Journal 12 (2006): 5418–5427, 10.1002/chem.200501076. 16514689

[30] A. G. Doyle and E. N. Jacobsen , “Small‐Molecule H‐Bond Donors in Asymmetric Catalysis,” Chemical Reviews 107 (2007): 5713–5743, 10.1021/cr068373r. 18072808

[31] R. R. Knowles and E. N. Jacobsen , “Attractive Noncovalent Interactions in Asymmetric Catalysis: Links Between Enzymes and Small Molecule Catalysts,” Proceedings of the National Academy of Sciences 107 (2010): 20678–20685, 10.1073/pnas.1006402107. PMC2996434 20956302

[32] P. R. Schreiner , “Metal‐Free Organocatalysis Through Explicit Hydrogen Bonding Interactions,” Chemical Society Reviews 32 (2003): 289–296, 10.1039/b107298f. 14518182

[33] Y. Takemoto , “Development of Chiral Thiourea Catalysts and Its Application to Asymmetric Catalytic Reactions,” Chemical and Pharmaceutical Bulletin 58 (2010): 593–601, 10.1248/cpb.58.593. 20460782

[34] C. Xu and C. C. J. Loh , “A Multistage Halogen Bond Catalyzed Strain‐Release Glycosylation Unravels New Hedgehog Signaling Inhibitors,” Journal of the American Chemical Society 141 (2019): 5381–5391, 10.1021/jacs.9b00040. 30848592

[35] C. S. Bennett and M. C. Galan , “Methods for 2‐Deoxyglycoside Synthesis,” Chemical Reviews 118 (2018): 7931–7985, 10.1021/acs.chemrev.7b00731. 29953219 PMC6135715

[36] S. Das , D. Pekel , J. M. Neudörfl , and A. Berkessel , “Organocatalytic Glycosylation by Using Electron‐Deficient Pyridinium Salts,” Angewandte Chemie International Edition 54 (2015): 12479–12483, 10.1002/anie.201503156. 26220811

[37] T. Ghosh , A. Mukherji , and P. K. Kancharla , “Sterically Hindered 2,4,6‐Tri‐ tert ‐butylpyridinium Salts as Single Hydrogen Bond Donors for Highly Stereoselective Glycosylation Reactions of Glycals,” Organic Letters 21 (2019): 3490–3495, 10.1021/acs.orglett.9b00626. 31050439

[38] E. I. Balmond , D. Benito‐Alifonso , D. M. Coe , R. W. Alder , E. M. McGarrigle , and M. C. Galan , “A 3,4‐trans‐Fused Cyclic Protecting Group Facilitates α‐Selective Catalytic Synthesis of 2‐Deoxyglycosides,” Angewandte Chemie International Edition 53 (2014): 8190–8194, 10.1002/anie.201403543. 24953049 PMC4499252

[39] E. I. Balmond , D. Benito‐Alifonso , D. M. Coe , R. W. Alder , E. M. McGarrigle , and M. C. Galan , “A 3,4‐trans‐Fused Cyclic Protecting Group Facilitates α‐Selective Catalytic Synthesis of 2‐Deoxyglycosides,” Angewandte Chemie 126 (2014): 8329–8333, 10.1002/ange.201403543. PMC4499252 24953049

[40] G. A. Bradshaw , A. C. Colgan , N. P. Allen , et al., “Stereoselective Organocatalyzed Glycosylations—Thiouracil, Thioureas and Monothiophthalimide Act as Brønsted Acid Catalysts at Low Loadings,” Chemical Science 10 (2019): 508–514, 10.1039/C8SC02788A. 30713648 PMC6334493

[41] C. Xu , V. U. B. Rao , J. Weigen , and C. C. J. Loh , “A Robust and Tunable Halogen Bond Organocatalyzed 2‐Deoxyglycosylation Involving Quantum Tunneling,” Nature Communications 11 (2020): 4911, 10.1038/s41467-020-18595-2. PMC7527348 32999276

[42] P. R. Schreiner , “Tunneling Control of Chemical Reactions: The Third Reactivity Paradigm,” Journal of the American Chemical Society 139 (2017): 15276–15283, 10.1021/jacs.7b06035. 29028320

[43] P. R. Schreiner , “Quantum Mechanical Tunneling Is Essential to Understanding Chemical Reactivity,” Trends in Chemistry 2 (2020): 980–989, 10.1016/j.trechm.2020.08.006.

[44] A. P. Hernandez and V. Mamane , “Chalcogen Bonding in Complexes Based on Electrophilic Tellurium,” Coordination Chemistry Reviews 551 (2026): 217416, 10.1016/j.ccr.2025.217416.

[45] W. Wang , Q. Song , and Y. Wang , “Design, Evolution, and Application of Halogen, Chalcogen, Pnictogen, and Tetrel Bonding Catalysts,” Coordination Chemistry Reviews 549 (2026): 217374, 10.1016/j.ccr.2025.217374.

[46] C. Zhao , D. Chen , Z. Wang , and Y. Zeng , “Chalcogen Bond in Organocatalysis: Bonding Characteristics, Catalytic Mechanisms, and Catalyst Design,” Coordination Chemistry Reviews 549 (2026): 217269, 10.1016/j.ccr.2025.217269.

[47] M. Mahanti and M. C. Galan , “Sigma Hole Donor XB and ChB Catalysis in Oligosaccharide Synthesis,” Trends in Chemistry 7 (2025): 571–575, 10.1016/j.trechm.2025.07.001.

[48] Z. Zhao and Y. Wang , “Chalcogen Bonding Catalysis With Phosphonium Chalcogenide (PCH),” Accounts of Chemical Research 56 (2023): 608–621, 10.1021/acs.accounts.3c00009. 36802469

[49] H. Barbero , C. Díez‐Poza , and A. Barbero , “The Oxepane Motif in Marine Drugs,” Marine Drugs 15 (2017): 361, 10.3390/md15110361. 29140270 PMC5706050

[50] S. Basu , B. Ellinger , S. Rizzo , et al., “Biology‐Oriented Synthesis of a Natural‐Product Inspired Oxepane Collection Yields a Small‐Molecule Activator of the Wnt‐Pathway,” Proceedings of the National Academy of Sciences 108 (2011): 6805–6810, 10.1073/pnas.1015269108. PMC3084053 21415367

[51] A. B. Nejma , A. Nguir , H. B. Jannet , et al., “New Septanoside and 20‐Hydroxyecdysone Septanoside Derivative From Atriplex Portulacoides Roots With Preliminary Biological Activities,” Bioorganic & medicinal chemistry letters 25 (2015): 1665–1670. 25813159 10.1016/j.bmcl.2015.03.028

[52] S. Castro , M. Duff , N. L. Snyder , M. Morton , C. V. Kumar , and M. W. Peczuh , “Recognition of Septanose Carbohydrates by Concanavalin A,” Organic & Biomolecular Chemistry 3 (2005): 3869–3872, 10.1039/b509243d. 16239999

[53] S. K. Jana , S. Harikrishna , S. Sudhakar , R. El‐Khoury , P. I. Pradeepkumar , and M. J. Damha , “Nucleoside Analogues With a Seven‐Membered Sugar Ring: Synthesis and Structural Compatibility in DNA–RNA Hybrids,” The Journal of Organic Chemistry 87 (2022): 2367–2379, 10.1021/acs.joc.1c02254. 35133166

[54] S. Manta and N. Kollatos , “Unusual Seven‐Membered Ring Sugars and Nucleosides: Synthesis and Biological Properties,” Nucleosides, Nucleotides & Nucleic Acids 42 (2023): 407–425, 10.1080/15257770.2022.2151623. 36451584

[55] D. A. Cruz , V. Sinka , V. S. Martín , and J. I. Padrón , “Iron‐Catalyzed Prins–Peterson Reaction for the Direct Synthesis of Δ 4‐2,7‐Disubstituted Oxepenes,” The Journal of Organic Chemistry 83 (2018): 12632–12647, 10.1021/acs.joc.8b01978. 30252471

[56] M. L. Lanier , A. C. Kasper , H. Kim , and J. Hong , “Synthesis of α,α′‐trans ‐Oxepanes Through an Organocatalytic Oxa‐conjugate Addition Reaction,” Organic Letters 16 (2014): 2406–2409, 10.1021/ol500773w. 24724535 PMC4018174

[57] M. W. Peczuh and N. L. Snyder , “Carbohydrate‐Based Oxepines: Ring Expanded Glycals for the Synthesis of Septanose Saccharides,” Tetrahedron Letters 44 (2003): 4057–4061, 10.1016/S0040-4039(03)00849-9.

[58] V. Sinka , D. A. Cruz , V. S. Martín , and J. I. Padrón , “Shortest Enantioselective Total Syntheses of (+)‐Isolaurepinnacin and (+)‐Neoisoprelaurefucin,” Organic Letters 24 (2022): 5271–5275, 10.1021/acs.orglett.2c01769. 35834432 PMC9344465

[59] J. O. Hoberg , “Formation of Seven‐Membered Oxacycles Through Ring Expansion of Cyclopropanated Carbohydrates,” The Journal of Organic Chemistry 62 (1997): 6615–6618, 10.1021/jo970649v.

[60] R. Batchelor and J. O. Hoberg , “Diastereoselective Formation of Seven‐Membered Oxacycles by Ring‐Expansion of Cyclopropanated Galactal,” Tetrahedron Letters 44 (2003): 9043–9045, 10.1016/j.tetlet.2003.09.222.

[61] W. Ma , J. L. Kirchhoff , C. Strohmann , B. Grabe , and C. C. J. Loh , “Cooperative Bifurcated Chalcogen Bonding and Hydrogen Bonding as Stereocontrolling Elements for Selective Strain‐Release Septanosylation,” Journal of the American Chemical Society 145 (2023): 26611–26622, 10.1021/jacs.3c06984. 38032866 PMC10722516

[62] W. Ma , A. Schmidt , C. Strohmann , and C. C. J. Loh , “Stereoselective Entry Into α,α′‐C‐Oxepane Scaffolds Through a Chalcogen Bonding Catalyzed Strain‐Release C ‐Septanosylation Strategy,” Angewandte Chemie International Edition 63 (2024): 202405706, 10.1002/anie.202405706. 38687567

[63] G. Bashiardes , V. Chaussebourg , G. Laverdan , and J. Pornet , “A New Synthesis of 1,3‐Dihydrobenzo[1,2]oxasiloles by a Novel Rearrangement of a Pentavalent Silicon Intermediate,” Chemical Communications 1 (2004): 122–123, 10.1039/b311804e. 14737362

[64] B. M. Trost and A. Bertogg , “Si‐Based Benzylic 1,4‐Rearrangement/Cyclization Reaction,” Organic Letters 11 (2009): 511–513, 10.1021/ol802289f. 19125665 PMC2640427

[65] N. Kerru , A. Singh‐Pillay , P. Awolade , and P. Singh , “Current Anti‐Diabetic Agents and Their Molecular Targets: A Review,” European Journal of Medicinal Chemistry 152 (2018): 436–488, 10.1016/j.ejmech.2018.04.061. 29751237

[66] T. S. Lin , Y. W. Liw , J. S. Song , et al., “Synthesis and Biological Evaluation of Novel C‐aryl d‐Glucofuranosides as Sodium‐Dependent Glucose Co‐Transporter 2 Inhibitors,” Bioorganic & Medicinal Chemistry 21 (2013): 6282–6291, 10.1016/j.bmc.2013.08.067. 24071445

[67] J. T. Cheng , C. Guo , W. J. Cui , et al., “Isolation of Two Rare *N‐*Glycosides From *Ginkgo biloba* and their Anti‐Inflammatory Activities,” Scientific Reports 10 (2020): 5994. 32265463 10.1038/s41598-020-62884-1 PMC7138816

[68] S. Lakhdar , M. Westermaier , F. Terrier , et al., “Nucleophilic Reactivities of Indoles,” The Journal of Organic Chemistry 71 (2006): 9088–9095, 10.1021/jo0614339. 17109534

[69] C. C. J. Loh and D. Enders , “Exploiting the Electrophilic Properties of Indole Intermediates: New Options in Designing Asymmetric Reactions,” Angewandte Chemie International Edition 51 (2012): 46–48, 10.1002/anie.201107575. 22162240

[70] H. Guo , J. L. Kirchhoff , C. Strohmann , B. Grabe , and C. C. J. Loh , “Exploiting π and Chalcogen Interactions for the β‐Selective Glycosylation of Indoles Through Glycal Conformational Distortion,” Angewandte Chemie International Edition 63 (2024): 202316667, 10.1002/anie.202316667. 38116860

[71] G. Horne , F. X. Wilson , J. Tinsley , D. H. Williams , and R. Storer , “Iminosugars Past, Present and Future: Medicines for Tomorrow,” Drug Discovery Today 16 (2011): 107–118, 10.1016/j.drudis.2010.08.017. 20817006

[72] R. J. Nash , A. Kato , C. Y. Yu , and G. W. J. Fleet , “Iminosugars as Therapeutic Agents: Recent Advances and Promising Trends,” Future Medicinal Chemistry 3 (2011): 1513–1521, 10.4155/fmc.11.117. 21882944

[73] E. M. Sánchez‐Fernández , M. I. García‐Moreno , J. M. García Fernández , and C. O. Mellet , Small Molecule Drug Discovery: Methods, Molecules and Applications (Elsevier, 2019).

[74] B. E. Tyrrell , A. C. Sayce , K. L. Warfield , J. L. Miller , and N. Zitzmann , “Iminosugars: Promising Therapeutics for Influenza Infection,” Critical Reviews in Microbiology 43 (2017): 521–545, 10.1080/1040841X.2016.1242868. 27931136 PMC5470110

[75] C. Wang , A. Krupp , C. Strohmann , B. Grabe , and C. C. J. Loh , “Harnessing Multistep Chalcogen Bonding Activation in the α‐Stereoselective Synthesis of Iminoglycosides,” Journal of the American Chemical Society 146 (2024): 10608–10620. 38564319 10.1021/jacs.4c00262 PMC11027159

[76] I. Herrera‐González , E. M. Sánchez‐Fernández , A. Sau , et al., “Stereoselective Synthesis of Iminosugar 2‐Deoxy(thio)glycosides From Bicyclic Iminoglycal Carbamates Promoted by Cerium(IV) Ammonium Nitrate and Cooperative Brønsted Acid‐Type Organocatalysis,” The Journal of Organic Chemistry 85 (2020): 5038–5047, 10.1021/acs.joc.0c00324. 32159355

[77] H. Guo and C. C. J. Loh , “Enhanced Selectivity of Chalcogen Bonding Over Halogen Bonding Catalyzed C‐Glycosylation Through Differentiated Intermediate Activation,” Angewandte Chemie International Edition 65 (2026): 17553, 10.1002/anie.202517553. PMC12790375 41230956

[78] D. J. Gorelik , S. P. Desai , S. Jdanova , J. A. Turner , and M. S. Taylor , “Transformations of Carbohydrate Derivatives Enabled by Photocatalysis and Visible Light Photochemistry,” Chemical Science 15 (2024): 1204–1236, 10.1039/D3SC05400D. 38274059 PMC10806712

[79] Y. Jiang , Y. Zhang , B. C. Lee , and M. J. Koh , “Diversification of Glycosyl Compounds via Glycosyl Radicals,” Angewandte Chemie International Edition 62 (2023): 202305138. 10.1002/anie.202305138 37278303

[80] W. Shang and D. Niu , “Radical Pathway Glycosylation Empowered by Bench‐Stable Glycosyl Donors,” Accounts of Chemical Research 56 (2023): 2473–2488, 10.1021/acs.accounts.3c00374. 37594017

[81] A. Shatskiy , E. V. Stepanova , and M. D. Kärkäs , “Exploiting Photoredox Catalysis for Carbohydrate Modification Through C–H and C–C Bond Activation,” Nature Reviews Chemistry 6 (2022): 782–805, 10.1038/s41570-022-00422-5. 37118094

[82] Y. Wei , L. Q. H. Lin , B. C. Lee , and M. J. Koh , “Recent Advances in First‐Row Transition Metal‐Catalyzed Reductive Coupling Reactions for π‐Bond Functionalization and C‐Glycosylation,” Accounts of Chemical Research 56 (2023): 3292–3312, 10.1021/acs.accounts.3c00531. 37917928

[83] C. C. J. Loh , “Synergistic catalysis: An emerging concept for selective carbohydrate synthesis,” Chem Catalysis 4 (2024): 100891.

[84] R. Z. Mao , F. Guo , D. C. Xiong , Q. Li , J. Duan , and X. S. Ye , “Photoinduced C–S Bond Cleavage of Thioglycosides and Glycosylation,” Organic Letters 17 (2015): 5606–5609, 10.1021/acs.orglett.5b02823. 26540490

[85] R. Z. Mao , D. C. Xiong , F. Guo , Q. Li , J. Duan , and X. S. Ye , “Light‐Driven Highly Efficient Glycosylation Reactions,” Organic Chemistry Frontiers 3 (2016): 737–743, 10.1039/C6QO00021E.

[86] K. M. Liu , P. Y. Wang , Z. Y. Guo , et al., “Iterative Synthesis of 2‐Deoxyoligosaccharides Enabled by Stereoselective Visible‐Light‐Promoted Glycosylation,” Angewandte Chemie International Edition 61 (2022): 202114726, 10.1002/anie.202114726. 35133053

[87] Q. D. Dang , Y. H. Deng , T. Y. Sun , et al., “Catalytic Glycosylation for Minimally Protected Donors and Acceptors,” Nature 632 (2024): 313–319, 10.1038/s41586-024-07695-4. 38885695

[88] Y. Jiang , Y. Wei , Q. Y. Zhou , et al., “Direct Radical Functionalization of Native Sugars,” Nature 631 (2024): 319–327, 10.1038/s41586-024-07548-0. 38898275 PMC11236704

[89] T. R. Li , C. Das , G. Piccini , and K. Tiefenbacher , “Tetrafluororesorcin[4]arene Hexameric Capsule Enables the Expansion of the Reactivity Space in Supramolecular Catalysis,” Journal of the American Chemical Society 147 (2025): 11108–11116, 10.1021/jacs.4c17029. 39908571

[90] T. R. Li , F. Huck , G. Piccini , and K. Tiefenbacher , “Mimicry of the Proton Wire Mechanism of Enzymes Inside a Supramolecular Capsule Enables β‐Selective O‐Glycosylations,” Nature Chemistry 14 (2022): 985–994, 10.1038/s41557-022-00981-6. 35798949

[91] T. R. Li , G. Piccini , and K. Tiefenbacher , “Supramolecular Capsule‐Catalyzed Highly β‐Selective Furanosylation Independent of the S N 1/S N 2 Reaction Pathway,” Journal of the American Chemical Society 145 (2023): 4294–4303, 10.1021/jacs.2c13641. 36751707

[92] D. Schmid , T. R. Li , B. Goldfuss , and K. Tiefenbacher , “Exploring the Glycosylation Reaction Inside the Resorcin[4]arene Capsule,” The Journal of Organic Chemistry 88 (2023): 14515–14526, 10.1021/acs.joc.3c01547. 37796244

[93] P. Wang , H. Huang , Q. Cai , et al., “Stereo‐Switchable Electrochemical Glycosylation,” Journal of the American Chemical Society 148 (2026): 16860–16871, 10.1021/jacs.6c00007. 41957899

[94] M. Liu , K. M. Liu , D. C. Xiong , et al., “Stereoselective Electro‐2‐Deoxyglycosylation From Glycals,” Angewandte Chemie International Edition 59 (2020): 15204–15208, 10.1002/anie.202006115. 32394599

